# 3-Ethyl-6-{1-[4-(2-methyl­prop­yl)phen­yl]eth­yl}-1,2,4-triazolo[3,4-*b*][1,3,4]thia­diazole

**DOI:** 10.1107/S1600536808027086

**Published:** 2008-08-30

**Authors:** Hoong-Kun Fun, Samuel Robinson Jebas, K. V. Sujith, P. S. Patil, B. Kalluraya

**Affiliations:** a X-ray Crystallography Unit, School of Physics, Universiti Sains Malaysia, 11800 USM, Penang, Malaysia; b Department of Studies in Chemistry, Mangalore University, Mangalagangotri, Mangalore 574199, India; c Department of Physics, K.L.E. Society’s K.L.E. Institute of Technology, Gokul Road, Hubli 590030, India

## Abstract

In the mol­ecule of the title compound, C_17_H_22_N_4_S, the triazolothia­diazole ring system is essentially planar and forms a dihedral angle of 74.34 (6)° with the benzene ring. In the crystal structure, mol­ecules are linked into chains running along the *b* axis by C—H⋯π inter­actions; adjacent chains are cross-linked *via* C—H⋯N hydrogen bonds and short S⋯N contacts [3.2694 (14) and 3.2953 (14) Å].

## Related literature

For a related structure, see: Fun *et al.* (2008 ▶). For biological activities of triazole and 1,3,4-thia­diazo­les, see: Al-Soud *et al.* (2004 ▶); Labanauskas *et al.* (2004 ▶); Mathew *et al.* (2006 ▶); Ragenovic *et al.* (2001 ▶). For pharmacological activities of thia­diazo­les, see: Karegoudar *et al.* (2008 ▶); Swamy *et al.* (2006 ▶); Wang *et al.* (1996 ▶). For the preparation, see: Bhalerao *et al.* (1994 ▶). For bond-length data, see: Allen *et al.* (1987 ▶).
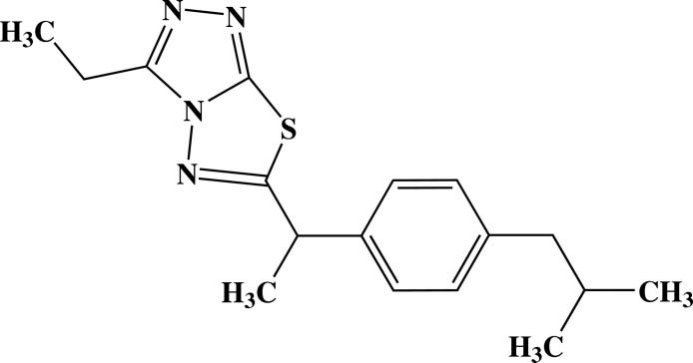


## Experimental

### 

#### Crystal data

C_17_H_22_N_4_S
*M* *_r_* = 314.45
Orthorhombic, 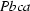

*a* = 11.4341 (5) Å
*b* = 9.1939 (4) Å
*c* = 30.9870 (13) Å
*V* = 3257.5 (2) Å^3^
*Z* = 8
Mo *K*α radiation
μ = 0.20 mm^−1^
*T* = 100.0 (1) K
0.44 × 0.09 × 0.05 mm

#### Data collection

Bruker SMART APEXII CCD area-detector diffractometer
Absorption correction: multi-scan (*SADABS*; Bruker, 2005 ▶) *T*_min_ = 0.917, *T*_max_ = 0.990
40117 measured reflections
5793 independent reflections
4079 reflections with *I* > 2σ(*I*)
*R*_int_ = 0.048

#### Refinement

*R*[*F* ^2^ > 2σ(*F* ^2^)] = 0.055
*wR*(*F* ^2^) = 0.135
*S* = 1.04
5793 reflections
203 parameters
H-atom parameters constrained
Δρ_max_ = 0.60 e Å^−3^
Δρ_min_ = −0.49 e Å^−3^

### 

Data collection: *APEX2* (Bruker, 2005 ▶); cell refinement: *APEX2*; data reduction: *SAINT* (Bruker, 2005 ▶); program(s) used to solve structure: *SHELXTL* (Sheldrick, 2008 ▶); program(s) used to refine structure: *SHELXTL*; molecular graphics: *SHELXTL*; software used to prepare material for publication: *SHELXTL* and *PLATON* (Spek, 2003 ▶).

## Supplementary Material

Crystal structure: contains datablocks global, I. DOI: 10.1107/S1600536808027086/ci2662sup1.cif

Structure factors: contains datablocks I. DOI: 10.1107/S1600536808027086/ci2662Isup2.hkl

Additional supplementary materials:  crystallographic information; 3D view; checkCIF report

## Figures and Tables

**Table 1:** Hydrogen-bond geometry (Å, °)

*D*—H⋯*A*	*D*—H	H⋯*A*	*D*⋯*A*	*D*—H⋯*A*
C10—H10*A*⋯N1^i^	0.93	2.51	3.440 (2)	177
C15—H15*A*⋯*Cg*1^ii^	0.96	2.67	3.5090 (17)	146
C16—H16*B*⋯*Cg*1^iii^	0.97	2.93	3.6772 (18)	135
C17—H17*B*⋯*Cg*2^iii^	0.96	2.92	3.614 (2)	130
C15—H15*B*⋯*Cg*3^ii^	0.96	2.74	3.6335 (17)	155

Symmetry codes: (i) 
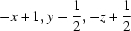
; (ii) 
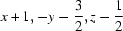
; (iii) 
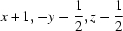
. *Cg*1, *Cg*2 and *Cg*3 are the centroids of the S1/C1/N3/N4/C3, N1/N2/C1/N3/C2 and C5–C10 rings, respectively.

**Fig. 1.**
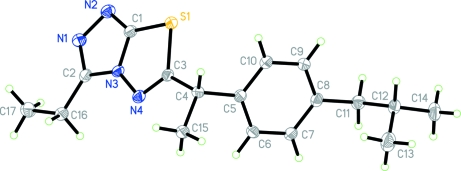
The molecular structure of the title compound, showing 50% probability displacement ellipsoids and the atom numbering scheme.

**Fig. 2.**
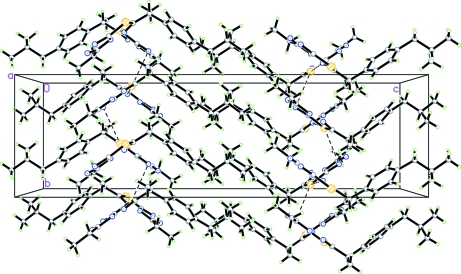
The crystal packing of the title compound, viewed along the a axis. Short S···N contacts are shown as dashed lines.

## supplementary crystallographic information

### Comment

1,2,4-Triazole and 1,3,4-thiadiazoles represent one of the most biologically active classes of compounds, possessing a wide spectrum of activities (Ragenovic *et al.*, 2001; Al-Soud *et al.*, 2004; Labanauskas *et al.*, 2004). Various substituted 1,2,4-triazolo[3,4-*b*]-1,3,4-thiadiazoles and their analogues are associated with diverse pharmacological activities such as antimicrobial (Swamy *et al.*, 2006), antibacterial (Wang *et al.*, 1996), antitubercular, anti-inflammatory and antifungal (Karegoudar *et al.*, 2008). A triazolo-thiadiazole system may be viewed as a cyclic analogue of two very important components viz. thiosemicarbazide and biguanide, which often display diverse biological activities (Mathew *et al.*, 2006). The required 4-amino-3-mercapto-5-ethyl-1,2,4-triazole was prepared in good yield through multi step reaction by using the method of Reid and Heindel (Bhalerao *et al.*, 1994). Phosphorous oxychloride was necessary for this condensation, which activate the carbonyl group of aromatic acids and increases its electrophilicity to enhance the addition of triazole to it. Previously, we have reported the crystal structure of a triazolo-thiadiazole system carrying ibuprofen moiety (Fun *et al.*, 2008). In continuation of our work, we report here the crystal structure of the title compound.

Bond lengths in the title molecule have normal values (Allen *et al.*, 1987). The triazolothiadiazole (S1/C1/N2/N1/C2/N3/N4/C3) ring system is essentially planar, with a maximium deviation of 0.013 (1) Å for atom S1. The dihedral angle between the triazolothiadiazole ring system and the benzene ring (C5–C10) is 74.34 (6)°.

The crystal packing is consolidated by weak C—H···π interactions (Table 1) involving the thiadiazole (S1/C1/N3/N4/C3, centroid Cg1), triazole (N1/N2/C1/N3/C2, centroid Cg2) and benzene (C5–C10, centroid Cg3) rings, and C—H···N hydrogen bonds. The C—H···π interactions link the molecules into chains running along the *b* axis. The adjacent chains are cross-linked via C—H···N hydrogen bonds (Table 1) and short S···N contacts [3.2694 (14) Å or 3.2953 (14) Å] (Fig. 2).

### Experimental

A mixture of 4-amino-3-mercapto-5-ethyl-1,2,4-triazole (0.01 mol), 2-(4-isobutylphenyl)propanoic acid (0.01 mol) and POCl_3_ (10 ml) was refluxed in a water bath for 16 h. Excess POCl_3_ was removed under reduced pressure. The reaction mixture was cooled, poured into crushed ice, and neutralized with sodium bicarbonate solution. The resulting solid product was filtered off, washed with water, dried, and recrystallized from ethanol-dimethylformamide (1/1, *v*/*v*) (yield 49%; m.p. 369–371 K). Analysis (%) for C_17_H_22_N_4_S found (calculated): C 64.89 (64.96), H 6.97 (7.006), N 17.78 (17.83) S 10.11 (10.19).

### Refinement

H atoms were positioned geometrically [C–H = 0.93-0.98 Å) and refined using a riding model, with *U*_iso_(H) = 1.2-1.5*U*_eq_ (C). A rotating-group model was used for the methyl groups.

### Crystal data

**Table:**

C_17_H_22_N_4_S	*F*_000_ = 1344
*M**_r_* = 314.45	*D*_x_ = 1.282 Mg m^−^^3^
Orthorhombic, *P**b**c**a*	Mo *K*α radiation λ = 0.71073 Å
Hall symbol: -P 2ac 2ab	Cell parameters from 8453 reflections
*a* = 11.4341 (5) Å	θ = 2.2–29.2º
*b* = 9.1939 (4) Å	µ = 0.20 mm^−^^1^
*c* = 30.9870 (13) Å	*T* = 100.0 (1) K
*V* = 3257.5 (2) Å^3^	Needle, colourless
*Z* = 8	0.44 × 0.09 × 0.05 mm

### Data collection

**Table:**

Bruker SMART APEXII CCD area-detector diffractometer	5793 independent reflections
Radiation source: fine-focus sealed tube	4079 reflections with *I* > 2σ(*I*)
Monochromator: graphite	*R*_int_ = 0.048
*T* = 100.0(1) K	θ_max_ = 32.4º
φ and ω scans	θ_min_ = 2.2º
Absorption correction: multi-scan(SADABS; Bruker, 2005)	*h* = −17→17
*T*_min_ = 0.917, *T*_max_ = 0.990	*k* = −13→13
40117 measured reflections	*l* = −40→46

### Refinement

**Table:**

Refinement on *F*^2^	Secondary atom site location: difference Fourier map
Least-squares matrix: full	Hydrogen site location: inferred from neighbouring sites
*R*[*F*^2^ > 2σ(*F*^2^)] = 0.055	H-atom parameters constrained
*wR*(*F*^2^) = 0.135	*w* = 1/[σ^2^(*F*_o_^2^) + (0.0517*P*)^2^ + 2.0837*P*] where *P* = (*F*_o_^2^ + 2*F*_c_^2^)/3
*S* = 1.04	(Δ/σ)_max_ = 0.001
5793 reflections	Δρ_max_ = 0.60 e Å^−^^3^
203 parameters	Δρ_min_ = −0.49 e Å^−^^3^
Primary atom site location: structure-invariant direct methods	Extinction correction: none

### Special details

**Table:**

Experimental. The data was collected with the Oxford Cyrosystem Cobra low-temperature attachment.
Geometry. All e.s.d.'s (except the e.s.d. in the dihedral angle between two l.s. planes) are estimated using the full covariance matrix. The cell e.s.d.'s are taken into account individually in the estimation of e.s.d.'s in distances, angles and torsion angles; correlations between e.s.d.'s in cell parameters are only used when they are defined by crystal symmetry. An approximate (isotropic) treatment of cell e.s.d.'s is used for estimating e.s.d.'s involving l.s. planes.
Refinement. Refinement of *F*^2^ against ALL reflections. The weighted *R*-factor *wR* and goodness of fit *S* are based on *F*^2^, conventional *R*-factors *R* are based on *F*, with *F* set to zero for negative *F*^2^. The threshold expression of *F*^2^ > σ(*F*^2^) is used only for calculating *R*-factors(gt) *etc*. and is not relevant to the choice of reflections for refinement. *R*-factors based on *F*^2^ are statistically about twice as large as those based on *F*, and *R*- factors based on ALL data will be even larger.

### Fractional atomic coordinates and isotropic or equivalent isotropic displacement parameters (Å^2^)

**Table:**

	*x*	*y*	*z*	*U*_iso_*/*U*_eq_	
S1	0.53939 (3)	0.06763 (4)	0.266708 (12)	0.01935 (10)	
N1	0.62584 (12)	0.31200 (15)	0.16919 (4)	0.0209 (3)	
N2	0.53144 (12)	0.24935 (15)	0.19196 (4)	0.0213 (3)	
N3	0.69861 (12)	0.18920 (14)	0.22283 (4)	0.0175 (3)	
N4	0.76251 (12)	0.11936 (15)	0.25470 (4)	0.0193 (3)	
C1	0.57968 (14)	0.17716 (17)	0.22372 (5)	0.0186 (3)	
C2	0.72430 (14)	0.27501 (17)	0.18787 (5)	0.0186 (3)	
C3	0.68928 (14)	0.05296 (16)	0.27988 (5)	0.0179 (3)	
C4	0.72469 (14)	−0.03276 (17)	0.31922 (5)	0.0178 (3)	
H4A	0.6795	−0.1233	0.3192	0.021*	
C5	0.69098 (14)	0.05277 (16)	0.35984 (5)	0.0182 (3)	
C6	0.76816 (15)	0.14778 (18)	0.38014 (5)	0.0206 (3)	
H6A	0.8439	0.1579	0.3696	0.025*	
C7	0.73313 (15)	0.22797 (18)	0.41606 (5)	0.0208 (3)	
H7A	0.7861	0.2906	0.4292	0.025*	
C8	0.62023 (15)	0.21602 (17)	0.43254 (5)	0.0194 (3)	
C9	0.54403 (15)	0.12017 (18)	0.41213 (5)	0.0204 (3)	
H9A	0.4683	0.1099	0.4226	0.025*	
C10	0.57850 (15)	0.03957 (17)	0.37645 (5)	0.0201 (3)	
H10A	0.5258	−0.0240	0.3635	0.024*	
C11	0.58366 (16)	0.29969 (18)	0.47213 (5)	0.0222 (3)	
H11A	0.5067	0.3408	0.4671	0.027*	
H11B	0.6377	0.3798	0.4763	0.027*	
C12	0.57973 (16)	0.20868 (18)	0.51362 (5)	0.0225 (3)	
H12A	0.5174	0.1363	0.5105	0.027*	
C13	0.69373 (19)	0.1279 (2)	0.52134 (6)	0.0376 (5)	
H13A	0.6888	0.0747	0.5479	0.056*	
H13B	0.7569	0.1964	0.5230	0.056*	
H13C	0.7075	0.0614	0.4980	0.056*	
C14	0.54985 (18)	0.3048 (2)	0.55214 (5)	0.0309 (4)	
H14A	0.5453	0.2462	0.5777	0.046*	
H14B	0.4759	0.3515	0.5473	0.046*	
H14C	0.6095	0.3773	0.5557	0.046*	
C15	0.85352 (14)	−0.07336 (18)	0.31744 (5)	0.0214 (3)	
H15A	0.8690	−0.1260	0.2913	0.032*	
H15B	0.8727	−0.1331	0.3418	0.032*	
H15C	0.9002	0.0134	0.3181	0.032*	
C16	0.84512 (15)	0.31316 (19)	0.17390 (5)	0.0223 (3)	
H16A	0.8872	0.2247	0.1669	0.027*	
H16B	0.8856	0.3602	0.1976	0.027*	
C17	0.84575 (17)	0.4136 (2)	0.13488 (6)	0.0279 (4)	
H17A	0.9250	0.4364	0.1272	0.042*	
H17B	0.8046	0.5016	0.1417	0.042*	
H17C	0.8080	0.3662	0.1110	0.042*	

### Atomic displacement parameters (Å^2^)

**Table:**

	*U*^11^	*U*^22^	*U*^33^	*U*^12^	*U*^13^	*U*^23^
S1	0.01693 (19)	0.02416 (19)	0.01697 (18)	0.00140 (16)	−0.00137 (14)	0.00051 (14)
N1	0.0194 (7)	0.0226 (6)	0.0208 (6)	0.0010 (6)	−0.0020 (5)	0.0008 (5)
N2	0.0206 (7)	0.0228 (6)	0.0204 (6)	0.0003 (6)	−0.0035 (5)	0.0018 (5)
N3	0.0152 (6)	0.0223 (6)	0.0150 (6)	0.0028 (5)	−0.0032 (5)	−0.0016 (5)
N4	0.0180 (7)	0.0236 (6)	0.0162 (6)	0.0037 (5)	−0.0028 (5)	0.0004 (5)
C1	0.0173 (7)	0.0207 (7)	0.0179 (7)	0.0010 (6)	−0.0022 (6)	−0.0016 (5)
C2	0.0201 (8)	0.0202 (7)	0.0156 (7)	0.0018 (6)	−0.0018 (6)	−0.0014 (5)
C3	0.0170 (7)	0.0216 (7)	0.0152 (6)	0.0025 (6)	−0.0012 (5)	−0.0031 (6)
C4	0.0157 (7)	0.0203 (7)	0.0174 (7)	−0.0008 (6)	−0.0005 (6)	0.0007 (5)
C5	0.0177 (8)	0.0213 (7)	0.0156 (7)	0.0016 (6)	−0.0016 (6)	0.0029 (6)
C6	0.0160 (8)	0.0273 (8)	0.0187 (7)	−0.0008 (6)	0.0009 (6)	0.0040 (6)
C7	0.0206 (8)	0.0243 (7)	0.0176 (7)	−0.0033 (6)	−0.0021 (6)	0.0005 (6)
C8	0.0213 (8)	0.0211 (7)	0.0159 (7)	0.0011 (6)	−0.0011 (6)	0.0009 (6)
C9	0.0169 (8)	0.0261 (7)	0.0183 (7)	−0.0009 (6)	0.0000 (6)	0.0000 (6)
C10	0.0199 (8)	0.0231 (7)	0.0173 (7)	−0.0015 (6)	−0.0019 (6)	−0.0001 (6)
C11	0.0229 (8)	0.0220 (7)	0.0216 (7)	0.0004 (7)	−0.0003 (6)	−0.0025 (6)
C12	0.0250 (8)	0.0251 (7)	0.0173 (7)	−0.0009 (7)	0.0011 (6)	−0.0027 (6)
C13	0.0396 (12)	0.0501 (12)	0.0232 (9)	0.0150 (10)	−0.0006 (8)	0.0033 (8)
C14	0.0390 (11)	0.0327 (9)	0.0209 (8)	−0.0027 (8)	0.0081 (7)	−0.0058 (7)
C15	0.0191 (8)	0.0254 (7)	0.0197 (7)	0.0004 (7)	−0.0011 (6)	0.0021 (6)
C16	0.0197 (8)	0.0283 (8)	0.0190 (7)	−0.0001 (7)	−0.0025 (6)	0.0018 (6)
C17	0.0250 (9)	0.0339 (9)	0.0250 (8)	−0.0023 (8)	−0.0005 (7)	0.0058 (7)

### Geometric parameters (Å, °)

**Table:**

S1—C1	1.7323 (16)	C9—H9A	0.93
S1—C3	1.7669 (16)	C10—H10A	0.93
N1—C2	1.311 (2)	C11—C12	1.535 (2)
N1—N2	1.412 (2)	C11—H11A	0.97
N2—C1	1.309 (2)	C11—H11B	0.97
N3—C1	1.365 (2)	C12—C13	1.519 (3)
N3—C2	1.372 (2)	C12—C14	1.524 (2)
N3—N4	1.3861 (17)	C12—H12A	0.98
N4—C3	1.297 (2)	C13—H13A	0.96
C2—C16	1.490 (2)	C13—H13B	0.96
C3—C4	1.507 (2)	C13—H13C	0.96
C4—C15	1.521 (2)	C14—H14A	0.96
C4—C5	1.533 (2)	C14—H14B	0.96
C4—H4A	0.98	C14—H14C	0.96
C5—C10	1.391 (2)	C15—H15A	0.96
C5—C6	1.392 (2)	C15—H15B	0.96
C6—C7	1.394 (2)	C15—H15C	0.96
C6—H6A	0.93	C16—C17	1.522 (2)
C7—C8	1.393 (2)	C16—H16A	0.97
C7—H7A	0.93	C16—H16B	0.97
C8—C9	1.391 (2)	C17—H17A	0.96
C8—C11	1.507 (2)	C17—H17B	0.96
C9—C10	1.388 (2)	C17—H17C	0.96
			
C1—S1—C3	87.95 (8)	C8—C11—H11A	108.7
C2—N1—N2	109.27 (13)	C12—C11—H11A	108.7
C1—N2—N1	105.09 (13)	C8—C11—H11B	108.7
C1—N3—C2	106.02 (13)	C12—C11—H11B	108.7
C1—N3—N4	118.26 (13)	H11A—C11—H11B	107.6
C2—N3—N4	135.73 (14)	C13—C12—C14	110.66 (15)
C3—N4—N3	107.84 (13)	C13—C12—C11	111.92 (15)
N2—C1—N3	111.33 (14)	C14—C12—C11	110.27 (14)
N2—C1—S1	139.54 (14)	C13—C12—H12A	108.0
N3—C1—S1	109.12 (11)	C14—C12—H12A	108.0
N1—C2—N3	108.30 (14)	C11—C12—H12A	108.0
N1—C2—C16	127.38 (14)	C12—C13—H13A	109.5
N3—C2—C16	124.30 (14)	C12—C13—H13B	109.5
N4—C3—C4	124.01 (14)	H13A—C13—H13B	109.5
N4—C3—S1	116.82 (12)	C12—C13—H13C	109.5
C4—C3—S1	119.17 (12)	H13A—C13—H13C	109.5
C3—C4—C15	111.06 (13)	H13B—C13—H13C	109.5
C3—C4—C5	109.16 (12)	C12—C14—H14A	109.5
C15—C4—C5	113.52 (13)	C12—C14—H14B	109.5
C3—C4—H4A	107.6	H14A—C14—H14B	109.5
C15—C4—H4A	107.6	C12—C14—H14C	109.5
C5—C4—H4A	107.6	H14A—C14—H14C	109.5
C10—C5—C6	118.29 (14)	H14B—C14—H14C	109.5
C10—C5—C4	119.44 (14)	C4—C15—H15A	109.5
C6—C5—C4	122.23 (14)	C4—C15—H15B	109.5
C5—C6—C7	120.70 (15)	H15A—C15—H15B	109.5
C5—C6—H6A	119.6	C4—C15—H15C	109.5
C7—C6—H6A	119.6	H15A—C15—H15C	109.5
C8—C7—C6	121.16 (15)	H15B—C15—H15C	109.5
C8—C7—H7A	119.4	C2—C16—C17	112.23 (14)
C6—C7—H7A	119.4	C2—C16—H16A	109.2
C9—C8—C7	117.63 (14)	C17—C16—H16A	109.2
C9—C8—C11	121.30 (15)	C2—C16—H16B	109.2
C7—C8—C11	121.02 (15)	C17—C16—H16B	109.2
C10—C9—C8	121.49 (15)	H16A—C16—H16B	107.9
C10—C9—H9A	119.3	C16—C17—H17A	109.5
C8—C9—H9A	119.3	C16—C17—H17B	109.5
C9—C10—C5	120.72 (15)	H17A—C17—H17B	109.5
C9—C10—H10A	119.6	C16—C17—H17C	109.5
C5—C10—H10A	119.6	H17A—C17—H17C	109.5
C8—C11—C12	114.32 (13)	H17B—C17—H17C	109.5
			
C2—N1—N2—C1	0.16 (17)	N4—C3—C4—C5	107.16 (17)
C1—N3—N4—C3	0.31 (18)	S1—C3—C4—C5	−72.12 (15)
C2—N3—N4—C3	179.77 (16)	C3—C4—C5—C10	85.48 (17)
N1—N2—C1—N3	−0.05 (17)	C15—C4—C5—C10	−150.03 (14)
N1—N2—C1—S1	−178.90 (15)	C3—C4—C5—C6	−92.33 (17)
C2—N3—C1—N2	−0.07 (17)	C15—C4—C5—C6	32.2 (2)
N4—N3—C1—N2	179.54 (13)	C10—C5—C6—C7	−0.3 (2)
C2—N3—C1—S1	179.14 (10)	C4—C5—C6—C7	177.54 (14)
N4—N3—C1—S1	−1.25 (17)	C5—C6—C7—C8	−0.3 (2)
C3—S1—C1—N2	−179.82 (19)	C6—C7—C8—C9	0.6 (2)
C3—S1—C1—N3	1.31 (11)	C6—C7—C8—C11	178.26 (15)
N2—N1—C2—N3	−0.20 (17)	C7—C8—C9—C10	−0.4 (2)
N2—N1—C2—C16	178.03 (15)	C11—C8—C9—C10	−178.00 (15)
C1—N3—C2—N1	0.17 (17)	C8—C9—C10—C5	−0.2 (2)
N4—N3—C2—N1	−179.34 (15)	C6—C5—C10—C9	0.6 (2)
C1—N3—C2—C16	−178.13 (15)	C4—C5—C10—C9	−177.35 (14)
N4—N3—C2—C16	2.4 (3)	C9—C8—C11—C12	75.4 (2)
N3—N4—C3—C4	−178.48 (13)	C7—C8—C11—C12	−102.21 (18)
N3—N4—C3—S1	0.82 (16)	C8—C11—C12—C13	52.6 (2)
C1—S1—C3—N4	−1.30 (13)	C8—C11—C12—C14	176.26 (15)
C1—S1—C3—C4	178.03 (12)	N1—C2—C16—C17	3.7 (2)
N4—C3—C4—C15	−18.7 (2)	N3—C2—C16—C17	−178.28 (14)
S1—C3—C4—C15	161.97 (11)		

### Hydrogen-bond geometry (Å, °)

**Table:**

*D*—H···*A*	*D*—H	H···*A*	*D*···*A*	*D*—H···*A*
C10—H10A···N1^i^	0.93	2.51	3.440 (2)	177
C15—H15A···Cg1^ii^	0.96	2.67	3.5090 (17)	146
C16—H16B···Cg1^iii^	0.97	2.93	3.6772 (18)	135
C17—H17B···Cg2^iii^	0.96	2.92	3.614 (2)	130
C15—H15B···Cg3^ii^	0.96	2.74	3.6335 (17)	155

Symmetry codes: (i) −*x*+1, *y*−1/2, −*z*+1/2; (ii) *x*+1, −*y*−3/2, *z*−1/2; (iii) *x*+1, −*y*−1/2, *z*−1/2.

## References

[bb1] Allen, F. H., Kennard, O., Watson, D. G., Brammer, L., Orpen, A. G. & Taylor, R. (1987). *J. Chem. Soc. Perkin Trans. 2*, pp. S1–S19.

[bb2] Al-Soud, Y. A., Al-Dweri, M. N. & Al-Masoudi, N. A. (2004). *Farmaco*, **59**, 775–783. 10.1016/j.farmac.2004.05.006 15474054

[bb3] Bhalerao, U. T., Muralikrishna, C. & Rani, B. R. (1994). *Tetrahedron*, **50**, 4019–4024.

[bb4] Bruker (2005). *APEX2*, *SAINT* and *SADABS* Bruker AXS Inc., Madison, Wisconsin, USA.

[bb5] Fun, H.-K., Jebas, S. R., Razak, I. A., Sujith, K. V., Patil, P. S., Kalluraya, B. & Dharmaprakash, S. M. (2008). *Acta Cryst.* E**64**, o1076–o1077. 10.1107/S1600536808013883 PMC2961563 21202594

[bb6] Karegoudar, P., Prasad, D. J., Ashok, M., Mahalinga, M., Poojary, B. & Holla, B. S. (2008). *Eur. J. Med. Chem.* **43**, 808–815. 10.1016/j.ejmech.2007.06.026 17804121

[bb7] Labanauskas, L., Udrenaite, E., Gaidelis, P. & Bruktus, A. (2004). *Farmaco*, **59**, 255–259. 10.1016/j.farmac.2003.11.002 15081342

[bb8] Mathew, V., Keshavayya, J. & Vaidya, V. P. (2006). *Eur. J. Med. Chem.* **41**, 1048–1058. 10.1016/j.ejmech.2006.03.018 16822595

[bb9] Ragenovic, K. C., Dimova, V., Kakurinov, V., Labor, D. & Molnar, A. B. (2001). *Molecules*, **6**, 815–824.

[bb10] Sheldrick, G. M. (2008). *Acta Cryst.* A**64**, 112–122. 10.1107/S0108767307043930 18156677

[bb11] Spek, A. L. (2003). *J. Appl. Cryst.* **36**, 7–13.

[bb12] Swamy, S. N., Basappa, B. S., Prabhuswamy, P. B., Doreswamy, B. H., Prasad, J. S. & Rangappa, K. S. (2006). *Eur. J. Med. Chem.* **41**, 531–538. 10.1016/j.ejmech.2005.12.009 16529848

[bb13] Wang, Z., You, T., Xu, Y., Haijian, S. & Haoxin, S. (1996). *Molecules*, **1**, 68–71.

